# Screen-Printable Functional Nanomaterials for Flexible and Wearable Single-Enzyme-Based Energy-Harvesting and Self-Powered Biosensing Devices

**DOI:** 10.1007/s40820-023-01045-1

**Published:** 2023-03-31

**Authors:** Kornautchaya Veenuttranon, Kanyawee Kaewpradub, Itthipon Jeerapan

**Affiliations:** 1 Center of Excellence for Trace Analysis and Biosensor, Prince of Songkla University, Hat Yai, Songkhla, 90110 Thailand; 2 Division of Physical Science, Faculty of Science, Prince of Songkla University, Hat Yai, Songkhla, 90110 Thailand; 3 Center of Excellence for Innovation in Chemistry, Faculty of Science, Prince of Songkla University, Hat Yai, Songkhla, 90110 Thailand

**Keywords:** **S**creen-printable nanocomposites, Glucose, Glucose oxidase, Biofuel cells, Self-powered biosensors, Flexible bioelectronics

## Abstract

**Abstract:**

Developing flexible bioelectronics is essential to the realization of artificial intelligence devices and biomedical applications, such as wearables, but their potential is limited by sustainable energy supply. An enzymatic biofuel cell (BFC) is promising for power supply, but its use is limited by the challenges of incorporating multiple enzymes and rigid platforms. This paper shows the first example of screen-printable nanocomposite inks engineered for a single-enzyme-based energy-harvesting device and a self-powered biosensor driven by glucose on bioanode and biocathode. The anode ink is modified with naphthoquinone and multiwalled carbon nanotubes (MWCNTs), whereas the cathode ink is modified with Prussian blue/MWCNT hybrid before immobilizing with glucose oxidase. The flexible bioanode and the biocathode consume glucose. This BFC yields an open circuit voltage of 0.45 V and a maximum power density of 266 μW cm^−2^. The wearable device coupled with a wireless portable system can convert chemical energy into electric energy and detect glucose in artificial sweat. The self-powered sensor can detect glucose concentrations up to 10 mM. Common interfering substances, including lactate, uric acid, ascorbic acid, and creatinine, have no effect on this self-powered biosensor. Additionally, the device can endure multiple mechanical deformations. New advances in ink development and flexible platforms enable a wide range of applications, including on-body electronics, self-sustainable applications, and smart fabrics.

**Graphic Abstract:**

**Supplementary Information:**

The online version contains supplementary material available at 10.1007/s40820-023-01045-1.

## Highlights

- Screen-printable functional nanocomposite inks are engineered for flexible, single-enzyme-based energy-harvesting, and self-powered biosensing devices.
- A BFC powered by the same biosubstrate (glucose) is developed to harvest energy in a biofluid model and act as a self-powered electrochemical glucose.
- Customized inks are advantageous in terms of integrating with flexible materials, which can be integrated with a wide range of wearables and soft bioelectronics.

**Supplementary Information:**

The online version contains supplementary material available at 10.1007/s40820-023-01045-1.

## Graphical abstract

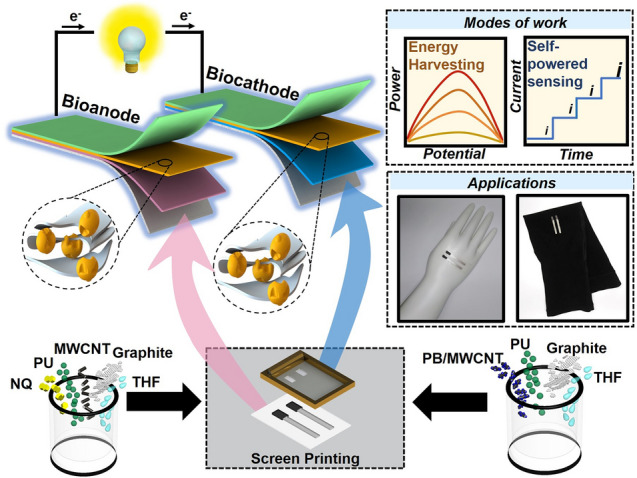

## Introduction

Flexible bioelectronics have many useful and versatile applications, such as wearables [1]. Unlike rigid, traditional platforms, a flexible device offers a unique opportunity to match the curvature of soft surfaces. For instance, modern on-body electronics, such as smart fabrics for tracking motion and monitoring biochemicals, are rapidly expanding. Soft robots and humanoid robots also benefit from flexible systems. Smart flexible devices open new opportunities for continuous fitness monitoring and are revolutionizing health care by enabling wellness monitoring. Regardless of the application, these devices should be fully integrated, lightweight, flexible, and autonomous from both a practical and esthetic perspective. To achieve the desired functions, it is necessary to develop several strategies. Further decreasing device size and fabrication costs are among them. To achieve this, incorporating customized materials and engineering ideas into a new design of flexible bioelectronics is essential.

A key barrier to the advancement of fully integrated flexible devices is the lack of energy. To overcome the energy requirements of soft devices, a common approach is to enhance the volumetric capacity and power density of flexible energy storage devices [2]. However, traditional systems still use rigid batteries without a self-charging technique; therefore, they must be periodically charged or replaced [3]. In addition to energy-storage systems, there is a growing interest in energy-harvesting technologies that convert surrounding energy (e.g., biomechanical, biochemical, and solar energy) into electricity. Motivated by the desire to realize the energy-sustainable concept, enzymatic biofuel cells (BFCs), which convert biochemical energy available in human biofluids into electricity, are among the most powerful alternatives for energy generation. This is due to their advantages for operation with enzymes that are active at ambient temperature and under mild physiological conditions, allowing on-body, implantable, and ingestible applications in biological systems [4–6].

Significantly, in addition to being energy-conversion devices, BFCs can also be used as self-powered electrochemical biosensors to sense analytes without external power [4]. Since the generated power is typically proportionate to the analyte concentration, BFCs can monitor the level of a specific substrate in real time as a stand-alone device. Enzymatic BFCs use enzymes (e.g., glucose oxidase (GOx)) as biocatalysts to convert the chemical energy of biofuels (e.g., glucose) in a body fluid into electrical energy. Thanks to the high selectivity of enzymes, it is not necessary to separate the anodic and cathodic counterparts, enabling the straightforward design of a two-electrode system that could be further developed into practical miniaturized devices. As of now, self-powered BFCs have been successfully used to detect a variety of chemicals, such as glucose [7], cholesterol [8], and lactate [9].

Even though substantial advances have been made in developing BFC-based energy harvesters or self-powered biosensors, most current technologies use BFCs with a two-enzyme configuration (using different enzymes on a bioanode and a biocathode). The development of bi-enzymatic BFCs faces critical challenges. First, different enzymes require different operating conditions, such as pH. There is a significant difference between laboratory applications using gold-standard setups with separation membranes and chambers and real-world applications. Managing pH is too challenging for wearable devices and miniaturized devices; it is hard to control one pH for the wearable anode while adjusting the other pH value for the cathode. Those factors can affect the BFC performance. In addition, the use of two enzymes complicates the BFC design and increases its cost. For example, GOx costs only 0.4 USD per 100 units, whereas laccase and bilirubin oxidase cost much more (30–700 USD per 100 units). Hence, we aim to address such grand challenges by engineering new BFCs with only one enzyme on the anode and the cathode.

Despite the advantages of wearable BFCs and the single-enzyme-BFC configuration, no reports exist today to demonstrate a single-enzyme BFC and self-powered biosensors on any flexible or printed platforms (Table S1). Previously, a BFC powered by the glucose for both the bioanode and the biocathode, yielding the maximum powder of 3.5 μW cm^−2^, was illustrated [10]. On this bioanode, GOx was immobilized on a modified graphite rod electrode. In fact, GOx was co-immobilized with an additional enzyme, i.e., horseradish peroxidase (HRP) on the rigid cathode. Note that the extra cost due to HRP (~ 13 USD per 100 unit) would increase the fabrication cost. Another BFC on rigid graphite rods was reported [7]. Both the bioanode and the biocathode were immobilized with the same enzyme (i.e., GOx), while the BFCs operated by GOx oxidation at the bioanode and H_2_O_2_ reduction at the biocathode. The power output of the system obtained only 10.9 μW cm^−2^. Notably, these BFC designs are limited by the use of rigid platforms, that are hard to integrate into biosystems. To the best of our knowledge there are no publications on any flexible single-enzyme BFCs that demonstrate the applicability in simulated biological fluids.

**Fig. 1:**
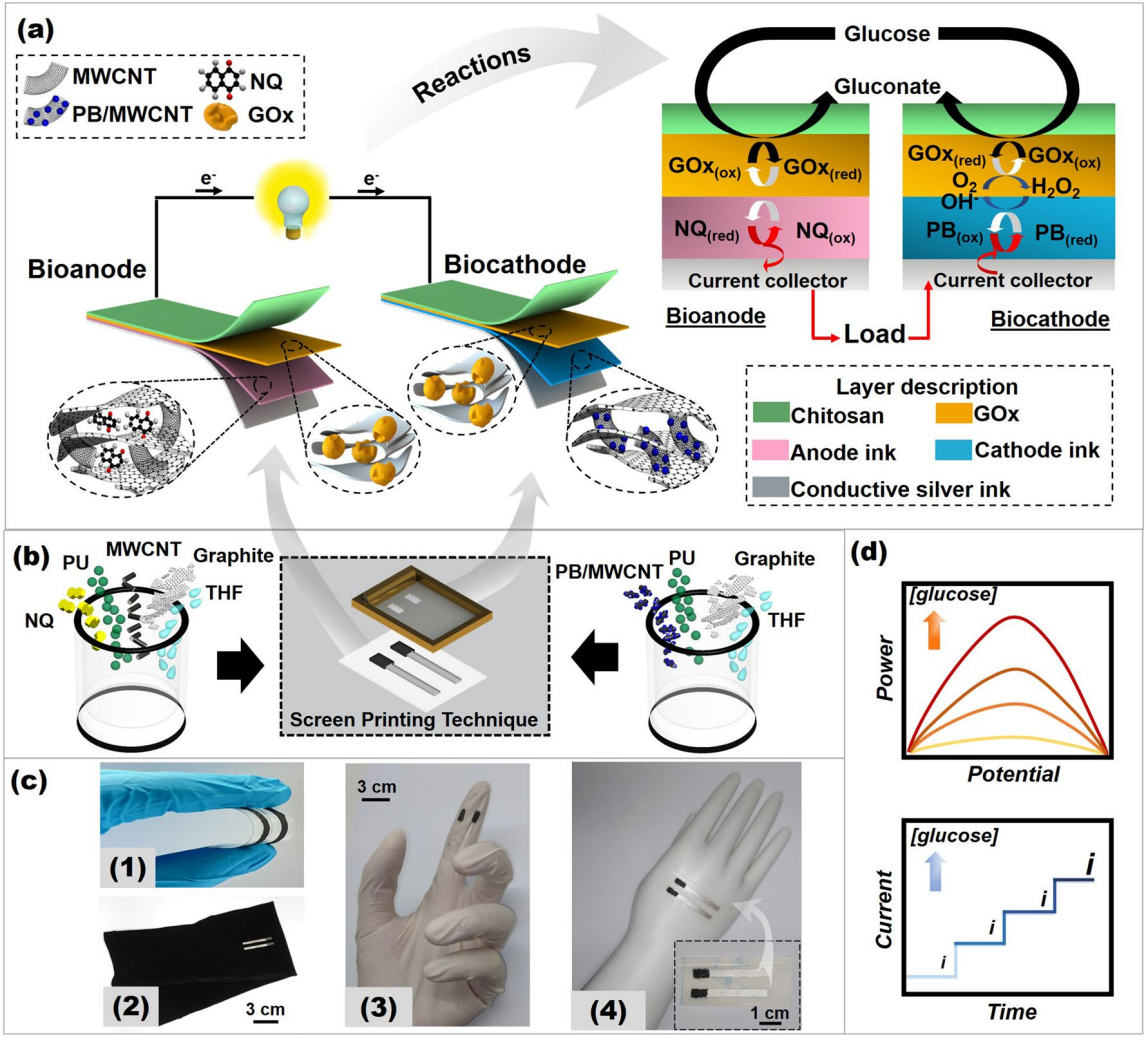
The conceptual presentation of a screen-printed and flexible single-enzyme-based system for harvesting energy from glucose and self-powered sensing glucose. **a** The components of a screen-printed glucose BFC along with redox reactions occurring on the bioanode and the biocathode. **b** Preparation of the screen-printable inks for the anode and the cathode. **c** Photographs of a screen-printed glucose BFC on (1) PET, (2) a stretchable textile (arm sleeve), (3) a glove (fingertip), and (4) a stretchable epidermal tattoo attached to a hand model. **d** The working operation of a screen-printed glucose BFC on (top and bottom) energy-harvesting and self-powered sensing modes

This paper presents the first example of screen-printable functional inks engineered for a flexible single-enzyme-based energy-harvesting device and a self-powered biosensor, which use only glucose on the bioanode and biocathode (Fig. 1). We developed new printable and highly flexible inks for both the anode and the cathode. In addition to demonstrating a single-enzyme-based BFC design, a key novelty of the present work is the customized formulations of nanocomposites. These nanocomposites have high conductivity and can adhere to a range of versatile substrates, including stretchable textile, plastic, stretchable epidermal tattoo, and rubber-based materials. The reactions occurring on the BFCs were driven by the same biofuel (glucose) for both the bioanode and the biocathode by employing the same biocatalyst (GOx) immobilized on the surface of both electrodes (Fig. 1a). At the bioanode, the electrocatalytic reaction involves glucose oxidation. This results from electron transfer between the GOx active site and 1,4-naphthoquinone (NQ)-mediated nanocomposite. At the biocathode that uses glucose to produce hydrogen peroxide (H_2_O_2_), the reaction is based on H_2_O_2_ reduction, aided by Prussian blue (PB) as an electrocatalyst. Thus, the combination of such oxidation and reduction reactions allows the formation of a single-enzyme-based device to harvest energy from glucose and act as a self-powered glucose biosensor. The successful application of these screen-printed BFCs to human sweat model suggests their potential as flexible, energy-sustaining, and noninvasive glucose monitoring devices.

## Experimental Section

To demonstrate the concept of self-powered BFC, a screen-printed GOx/NQ/MWCNT-based bioanode and a screen-printed GOx/PB/MWCNT-based biocathode were prepared, followed by morphology study, electrode characterization, resistance, and electrochemical measurement. The BFC characterization and self-powered detection were performed, followed by an application study such as selectivity and operational stability. Mechanical resiliency was investigated (see the Supporting Information for details). The chemicals, reagents and artificial sweat used in this study are also described in the Supporting Information.

## Results and Discussion

### Concept of a Screen-Printed Glucose BFC

The operation of our single-enzyme BFC is based on glucose oxidation catalyzed by GOx at the bioanode and H_2_O_2_ reduction catalyzed by PB-based nanocomposites at the biocathode (Fig. 1). Glucose was chosen as a model for both compartments because it is one of the most suitable biological substrates, commonly found in various biofluids, such as blood, tears, sweat, saliva, and interstitial fluids [11]. Our BFC converts glucose (a biological fuel) into electrical energy; this energy-conversion technology is different from conventional fuel cells, such as H_2_/O_2_ and methanol/O_2_ fuel cells, as they can operate under mild conditions, such as in physiological media and at ambient temperatures [4]. Additionally, a membrane is often required to separate anode and cathode compartments. The use of an enzymes (biocatalyst) in this work enables a membraneless BFC design due to the selectivity toward the specific biofuel. This meets our objective of simplifying BFC configurations.

At the bioanode, the oxidation reaction occurs when glucose interacts with the active site of GOx, resulting in electron transfer toward the printed nanocomposite-based electrode through NQ (a redox mediator) (Fig. 1a). These harvested electrons due to the glucose oxidation flow toward the load and the biocathode, completing the electrical circuit. Meanwhile, glucose is also used on the biocathode. First, GOx enzymatic reaction generates H_2_O_2_. This H_2_O_2_ along with gluconolactone are formed when glucose molecules reach the GOx immobilized on the biocathode in the presence of oxygen. The biocathode reaction is then based on H_2_O_2_ reduction with the help of printed PB-based nanomaterials as an electrocatalyst. Challengingly, when using a conventional electrode, H_2_O_2_ can be reduced only at a high overpotential [12]. Therefore, it is required to engineer nanocatalytic materials to decrease the overpotential of H_2_O_2_ cathodic reaction on the flexible biocathode.

Regarding the BFC fabrication, we engineered new screen-printable functional inks for supporting both the anode and the cathode to fabricate a highly flexible energy-harvesting device and a self-powered biosensor (Fig. 1b). We leveraged a screen-printing technique to fabricate the flexible electrodes because this technique is simple, high-throughput, scalable, and low-cost and can be applied to a wide range of substrate materials [13]. Importantly, inks can be modified to combine additional functions, such as electrochemical catalytic activities. Different inks and substrates can be used to create custom electrodes. To formulate highly flexible and homogeneous inks, chemical interactions between the added materials and mechanical force must be employed to aid in their preparation.

To improve the performance of the BFCs, it is necessary to add materials with extra functions to the ink recipes. The anode ink contains graphite and MWCNTs (as conductive fillers), NQ (as a redox mediator), and polyurethane (PU, as a polymeric binder) dispersed in an organic solvent (Fig. 1b, left). On the other side, the cathode ink comprises of graphite, a PB/MWCNT hybrid nanomaterial (as an electrocatalyst), and PU dispersed in tetrahydrofuran (THF) (Fig. 1b, right). Graphite and MWCNTs were used in both electrodes due to their good conductivity and stability [14, 15]. These inks contain graphite with a particle size less than 20 μm and MWCNTs with an aspect ratio of ~ 700–6000. These MWCNTs with a long tubular structure have a high specific surface area, allowing stable accommodation for GOx and NQ mediator molecules. NQ integrated in the MWCNTs matrix was used as redox mediator to shuttle electrons from the glucose oxidation at the mediated bioanode to enhance catalytic current densities and the adsorption of enzyme per unit volume [16]. Moreover, the non-covalent immobilization of NQ on graphite and MWCNTs matrix can be achieved by π−π interactions between the aromatic groups of quinones and MWCNTs/graphite surfaces, suppressing the NQ dissolution into liquid electrolyte and improving the electrode performance [17]. Regarding the cathode, PB/MWCNT hybrids were used as electrocatalysts because PB itself possesses a facile charge transfer [18]. The electrochemical characteristics of PB can be enhanced when it is combined with carbon-containing nanomaterials such as CNTs, graphene, and graphene oxide [19, 20]. Furthermore, the presence of MWCNTs in the ink can increase electron transfer between PB and the flexible electrode, thereby enhancing the electrochemical properties [21]. The π−π stacking can also be formed between the CNT sidewall and cyanide ligands of PB [22]. Since both carbon atoms in the sidewall of CNTs and the cyanide are conjugated, they could function as electron donor and acceptor, respectively. Therefore, high performances and stability of electrodes could be associated with the synergic effect between CNTs and PB [23].

A binder is essential to screen-printed ink’s flexibility, adhesion and mechanical robustness. However, applying an excessive amount of the binder can compromise the electrode characteristics and result in a non-conductive network. In this study, the ink recipes were properly customized to maintain conductive property and mechanical strength. PU was chosen as the binder because of its elastomeric properties and ability to reinforce the conductive network after the electrode has been mechanically distorted [24]. The desirable property of PU is due to various functional groups (e.g., amino and carbonyl groups) on PU molecular chains; thus, it not only improves adhesion properties between ink formulation and substrate, but also allows MWCNTs to disperse evenly and form a three-dimensional (3D) conductive network in the inks [24]. Moreover, the type of solvent used in ink preparation also significantly affects the ink homogeneity. In this work, THF was selected as a solvent, resulting in an optimal ink formulation with appropriate viscosity. This is because its dielectric constant is suitable to produce a colloidal-like ink, unlike other solvents (such as iso-propanol or ethylene glycol) which have a higher dielectric constant and produce a solvent-like ink [25].

It is necessary to agitate the agglomerates of solid materials during the dispersion of the ink components (containing solid contents and solvent) to enable the solid matter to be fully dispersed. In this work, the homogenizer probe was first used to disperse MWCNTs or PB/MWCNT nanocomposite in the solvent, then other contents were added and thoroughly mixed using a high-speed mixing machine. During the first step of dispersion, solid matter can be dispersed quickly by the homogenizer probe (less than 2 min). The use of a homogenizer probe to apply a mechanical shear force can breakdown the van de Waals potential energy between long nanotubes and can reduce the agglomerate diameter of solid contents resulting in more active sites. Reducing the agglomerate size can improve ink quality [26].

The ratio of ink components, which are conductive particles (MWCNTs and graphite), mediator (NQ), electrocatalyst (PB/MWCNT), polymer binder (PU), and solvent (THF), has been customized to realize good adhesion between ink formulations and substrates while maintaining high conductivity [27]. By squeezing the resulting ink through the opening areas of the stencil, electrode structures can be created on a wide range of surfaces and materials, including plastic, rubber, textile, and stretchable epidermal, as shown in Fig. 1c. Using a low curing temperature makes it possible to incorporate a variety of substrates without damaging them, making mass manufacturing of devices easier. Unlike other fabrication techniques, such as the light irradiation technique and electrostatic spray deposition, high temperatures may be required, rendering them incompatible with wearable substrates (such as textiles or soft thin films) because extreme conditions can damage the substrate surface [28].

Our BFC model utilizes the new ink formulations and bioelectrochemical reactions at the printed electrode surfaces to convert chemical energy into electrical energy, thereby generating current and power (Fig. 1d, top). This electrical energy from redox reactions is also proportional to fuel (glucose) concentration. Hence, the printed device can also act as a self-powered biosensor to determine the glucose concentration [4] (Fig. 1d, bottom).

###  Characterizations of the Anode

**Fig. 2:**
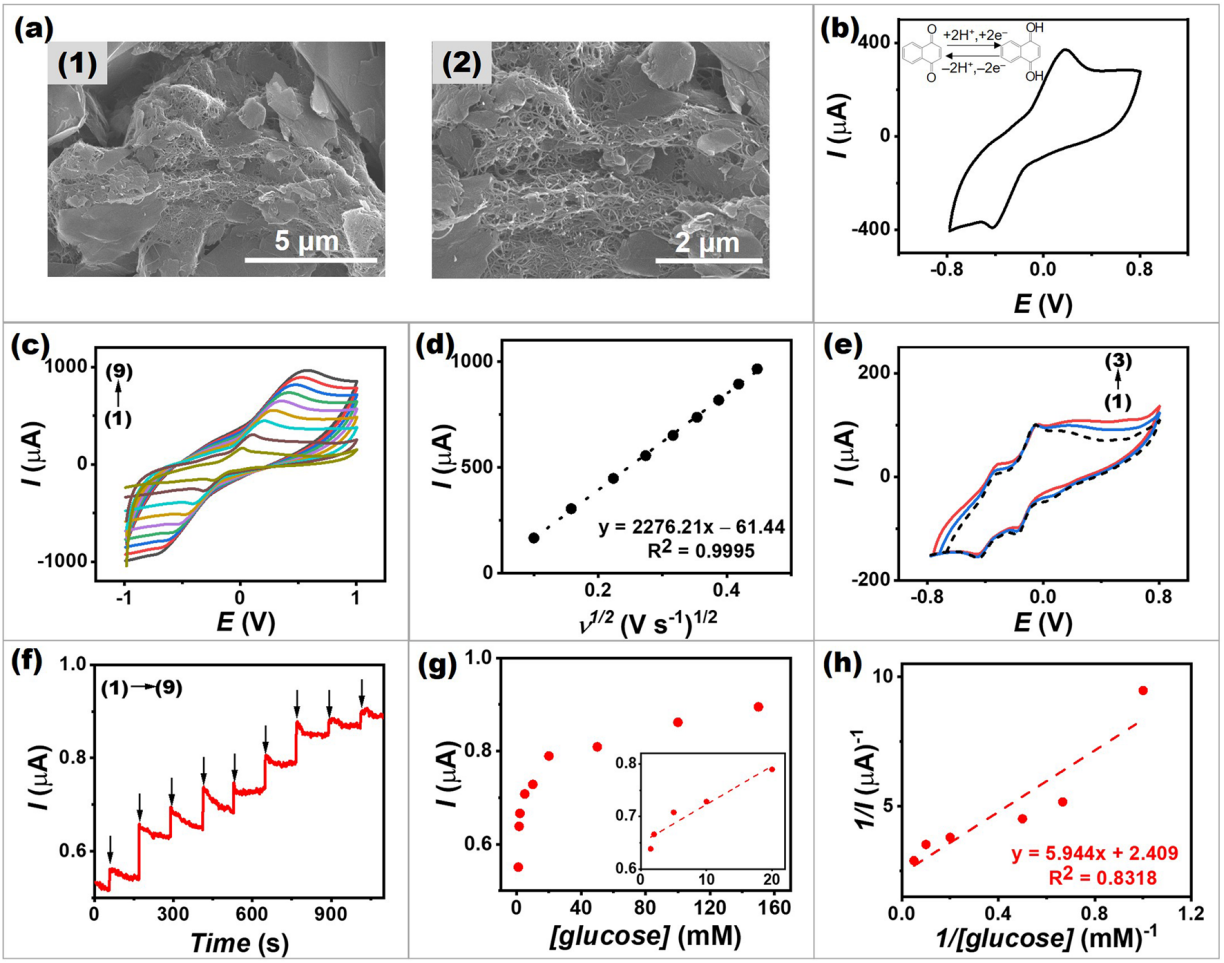
Characterizations of the anode. **a** SEM images of a screen-printed NQ/MWCNT-based anode at (1) low and (2) high magnification. **b** CV obtained from a screen-printed NQ/MWCNT-based anode in 0.1 M PBS, pH 7.0 with a scan rate of 50 mV s^−1^. **c** CVs obtained from a screen-printed NQ/MWCNT-based anode in 0.1 M PBS, pH 7.0 at different scan rates from 10 to 200 mV s^−1^ (scan rates (1–9): 10, 25, 50, 75, 100, 125, 150, 175 and 200 mV s^−1^). **d** Plots of anodic peak current densities in function with square root of the scan rate obtained on a screen-printed NQ/MWCNT-based anode. **e** CVs obtained from a screen-printed GOx/NQ/MWCNT-based bioanode in (1–3) 0, 10, and 100 mM glucose in 0.1 M PBS, pH 7.0 with a scan rate of 5 mV s^−1^. **f** Amperometric response of a screen-printed GOx/NQ/MWCNT-based bioanode with an applied potential of 0.3 V versus Ag/AgCl upon increasing the glucose concentrations ((1) 1.0 mM; (2) 1.5 mM; (3) 2.0 mM; (4) 5.0 mM; (5) 10 mM; (6) 20 mM; (7) 50 mM; (8) 100 mM; and (9) 150 mM). **g** The corresponding calibration plot of the current response of the GOx/NQ/MWCNT-based bioanode. **h** The double reciprocal plot of the calibration curve obtained from the GOx/NQ/MWCNT-based bioanode

To investigate morphology of the screen-printed NQ/MWCNT-based electrode, scanning electron microscopy (SEM) was carried out. As shown in Fig. 2a, MWCNTs and graphite particles were dispersed uniformly in the anode. On the surface of the printed electrode, components were evenly spread without significant aggregates. The network formed between compositions, particularly MWCNTs, creates good electrical connections. Moreover, a structure with high porosity enhances electrochemical properties due to increased active sites and good electron transfer, and can accommodate biocatalytic components [29].

A cyclic voltammetry (CV) with a three-electrode system was used to evaluate the electrochemical performance of NQ/MWCNT-based electrode. The CV curve at a scan rate of 50 mV s^−1^ illustrated in Fig. 2b shows that NQ embedded in the printed matrix displays a quasi-reversible redox capability, with a formal potential (*E*_1/2_) of − 0.12 V in pH 7.0. This redox couple was assigned to the two electron/two proton redox conversion [30]. The obvious redox peak in this study agreed with a previous study of an enzymatic electrode using NQ as an electron mediator [31].

Figure 2c shows the CVs of a screen-printed NQ/MWCNT-based anode obtained in 0.1 M PBS, pH 7.0, with a varying scan rate from 10 to 200 mV s^−1^. These plots indicate quasi-reversible NQ redox couples, and that the ratio of the anodic and cathodic peak currents (*I*_*c*_/*I*_*a*_ ratio) is nearly one (ranging from 0.9 to 1.0). The CV shapes are well maintained, indicating that the NQ/MWCNT-based electrode has good rate performance. The resulting anodic and cathodic peak currents are proportional to the square root of the scan rate, as shown in Fig. 2d. This behavior follows the Randles–Sevcik equation [32] and indicates that the processes occurring on the NQ/MWCNT-based anode are diffusion-controlled, which agrees with a previous study of a NQ-modified electrode [33].

GOx was immobilized to the NQ/MWCNT-based surface, and CV technique was performed at a low scan rate (5 mV s^−1^) to evaluate bioelectrocatalytic characteristics of the bioanode while minimizing the contribution of capacitive current caused by the large electroactive area of the ink’s matrix. As can be seen from voltammograms given in Fig. 2e (dashed line), a reversible peak was observed at around − 0.17 V in the absence of glucose. This redox behavior validates the immobilization of the enzyme and NQ on the surface of the bioanode. NQ could strongly functionalized in the inner matrix of the nanocomposite ink and some could be adsorbed at the surface, resulting in two overlapping reversible systems [34]. In the presence of glucose, the redox systems exhibit an increase in oxidation peak current at around 0 V while the reduction peak intensity decreases (Fig. 2e, solid line). This confirms that the electrochemical behavior of a screen-printed GOx/NQ/MWCNT-based bioanode upon adding glucose was due to an effective mediation by NQ and the enzyme immobilization incorporated in the conductive nano-matrix.

Furthermore, the electrocatalytic activity toward glucose oxidation on the GOx/NQ/MWCNT-based bioanode was investigated using an amperometric technique and increasing glucose concentrations (Fig. 2f). The result clearly shows that a rise in glucose concentration increases the anodic current. This is due to the effective contribution of the mediated glucose oxidation catalyzed by GOx. The current responses were observed over a wide range of 0–150 mM glucose before reaching its limit. As shown in Fig. 2g, the calibration plot of a single bioanode as a mediated glucose biosensor shows a linearity in a range of 1.5–20 mM glucose (*R*^2^ = 0.8988). The linear range sensitivity was calculated to be 0.0073 μA mM^−1^. Additionally, a Michaelis–Menten dependence is apparent, which corresponds to enzymatic kinetics. As illustrated in Fig. 2h, the graph was plotted in double reciprocal coordinates to transform the Michaelis–Menten equation (Eq. 1) algebraically into the Lineweaver–Burk equation (Eq. 2) [8]:

1 $$ I = \frac{{I_{\max } \left[ C \right]}}{{K_{m} + \left[ C \right]}} $$

2 $$ \frac{1}{I} = \frac{1}{{I_{\max } }} + \frac{{K_{m}^{{{\text{app}}}} }}{{I_{\max } }}\left( \frac{1}{C} \right) $$

where $I$ is the steady-state current after the addition of glucose, $I_{\max }$ is the maximum current obtained from saturated glucose concentrations, *C* is the glucose concentration, and $K_{m}^{{{\text{app}}}}$ is the apparent Michaelis–Menten constant (enzyme-substrate kinetic indicator). The $K_{m}^{{{\text{app}}}}$ interpreted from the straight line of the double reciprocal plot (using the slope and y-intercept) was 2.47 mM. It has a lower value than glucose biosensors based on glucose oxidation previously reported [16, 35]. The small value confirmed high affinity of the GOx immobilized on the bioanode to glucose.

###  Characterizations of the Cathode

**Fig. 3:**
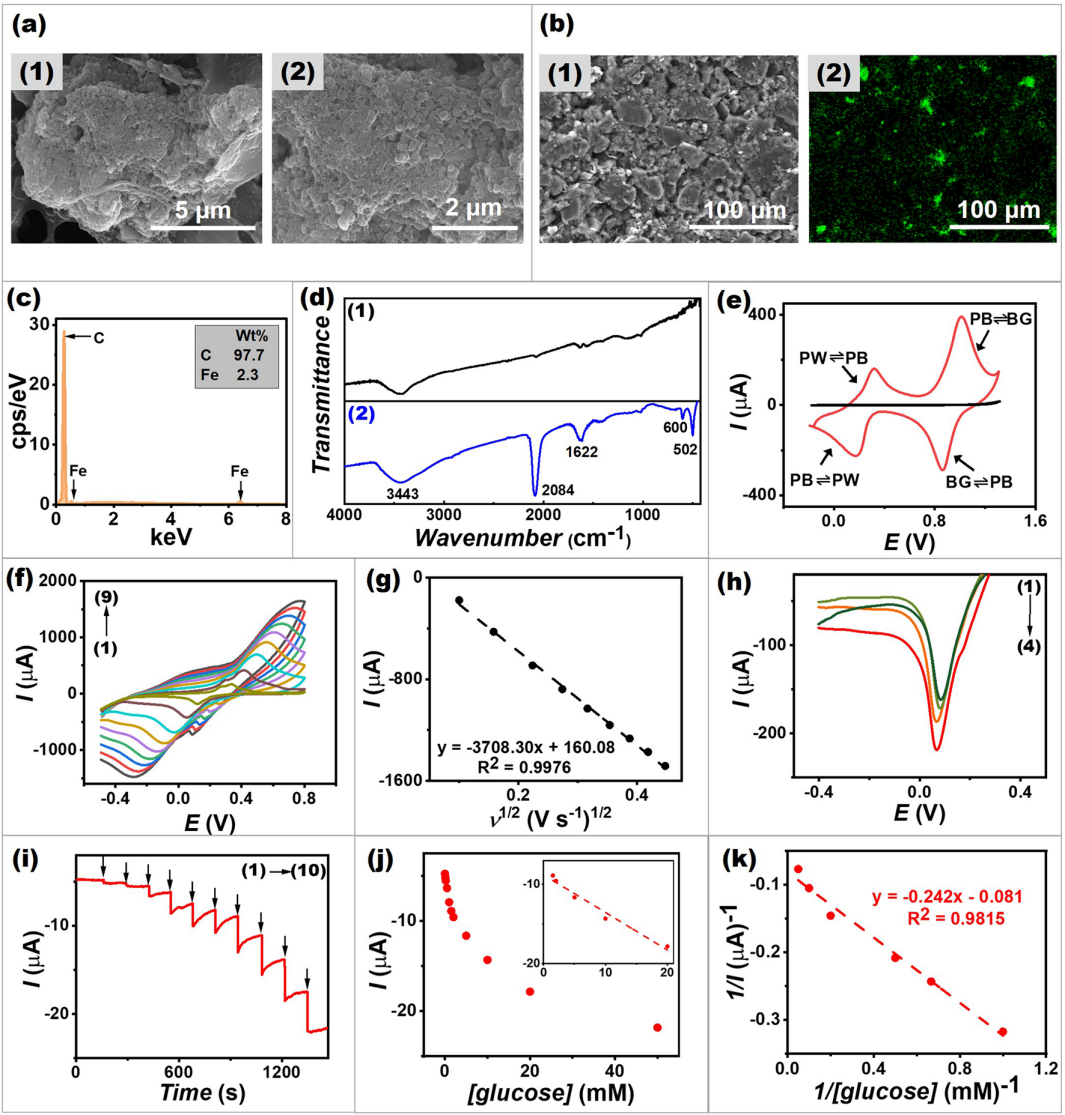
Characterizations of the cathode. **a** SEM images of a screen-printed PB/MWCNT-based cathode at (1) low magnification and (2) high magnification. **b** SEM image (1) with EDX mapping for (2) Fe elements in PB/MWCNT-based cathode. **c** EDX spectra of PB/MWCNT-based cathode. **d** FT-IR spectra of (1) MWCNT and (2) synthesized PB/MWCNT. **e** CVs obtained from (black line) the glassy carbon (GC) electrode and (red line) the PB/MWCNT-coated GC electrode in 0.1 M PBS, pH 7.0 with a scan rate of 50 mV s^−1^. **f** CVs obtained from a screen-printed PB/MWCNT-based cathode in 0.1 M PBS, pH 7.0 at different scan rates from 10 to 200 mV s^−1^ (scan rates (1–9): 10, 25, 50, 75, 100, 125, 150, 175 and 200 mV s^−1^). **g** Plots of cathodic peak current densities in function with square root of the scan rate obtained on a screen-printed PB/MWCNT-based cathode. **h** LSVs obtained from a screen-printed PB/MWCNT-based cathode in the presence of H_2_O_2_ ((1) blank; (2) 0.5 mM; (3) 2 mM; and (4) 5 mM) in 0.1 M PBS, pH 7.0 with a scan rate of 5 mV s^−1^. **i** Amperometric response of a screen-printed GOx/PB/MWCNT-based biocathode with an applied potential of 0 V versus Ag/AgCl upon increasing the glucose concentrations ((1) 0.1 mM; (2) 0.2 mM; (3) 0.5 mM; (4) 1 mM; (5) 1.5 mM; (6) 2 mM; (7) 5 mM; (8) 10 mM; (9) 20 mM; and (10) 50 mM). **j** The corresponding calibration plot of the current response of the GOx/PB/MWCNT-based biocathode. **k** The double reciprocal plot of the calibration curve obtained from the GOx/PB/MWCNT-based biocathode. (Color figure online)

The morphology of a screen-printed PB/MWCNT-based cathode was characterized by SEM (Fig. 3a). The study reveals uniform PB nanoparticles with an average particle diameter of ~ 250 nm at the cathode surface. This structure results from the nucleation and growth of PB particles during PB synthesis [36]. The small rod of MWCNTs was dispersed uniformly between each PB nanoparticles attached to graphite surface, thus ensuring the good conductivity within the continuous conductive network [36].

Figure 3b shows EDX elemental analysis within PB/MWCNT-based cathode. It is clearly seen in the EDX mapping that iron (Fe) elements are present and uniformly distributed in the cathode ink compound (Fig. 3b2). The presence of Fe is also validated by the EDX spectrum (Fig. 3c); the Fe peaks observed in the cathode composite confirmed the successful synthesis of PB. Moreover, FTIR was further used to characterize PB/MWCNT composite. The functional groups present in the synthesized PB/MWCNT assigned to H–O–H bend of water, C≡N stretch, O–H stretch of water, Fe–O bond and Fe^2+^–CN–Fe^3+^ bend with the absorption peak at 3443, 2084, 1622, 600, and 502 cm^−1^, respectively (Fig. 3d) [37, 38]. Note that the stretching vibration of C≡N and Fe^2+^–CN–Fe^3+^ bend are the typical signals of PB. − OH groups specifically at 3443 cm^−1^ are also found in MWCNTs; this may be introduced during the manufacturing process from raw materials [39]. The hydroxyl bending band specifically at 1622 cm^−1^ can be caused by MWCNT surface as well as an internal moisture left over from the PB synthesis process.

We also characterized the electrochemical behavior of the synthesized PB/MWCNT (Fig. 3e). Two redox pairs, clearly observed in the CV, were related to two reversible redox reactions: Prussian white (PW) ⇌ Prussian blue (PB) (redox reaction of Fe^3+/2+^) and Prussian blue (PB) ⇌ Berlin green (BG) (redox reaction of Fe(CN)_6_^3−/4−^) [40, 41]. Regarding the mechanism of H_2_O_2_ reduction on a PB-containing biocathode, PB can be electrochemically reduced to generate Prussian white (PW), which can catalyze the reduction of H_2_O_2_ at a low potential [42]. PW is then oxidized back to PB. This reversible electrochemical redox property of PB allows it to act as an efficient catalyst throughout the entire electrochemical reactions on the printed cathode.

Figure 3f shows the CVs of a screen-printed PB/MWCNT-based cathode obtained in 0.1 M PBS, pH 7.0, with varying scan rates from 10 to 200 mV s^−1^. These plots indicate quasi-reversible redox reaction of Fe^3+/2+^ in PB, and that the ratio of the anodic and cathodic peak currents (*I*_*c*_/*I*_*a*_ ratio) is nearly one (ranging from 0.9 to 1.1). The well-preserved CV shapes can be observed, and the values of *E*_pa_ and *E*_pc_ shift slightly to the positive and negative directions, respectively. The resulting anodic and cathodic peak currents are proportional to the square root of the scan rate, as shown in Fig. 3g. This behavior indicates that the processes occurring on the PB/MWCNT-based electrode are diffusion-controlled, which agrees with a previous study of a PB-modified electrode [42].

The electrochemical detection of H_2_O_2_ on a screen-printed PB/MWCNT-based cathode was investigated by a linear sweep voltammetry technique (LSV). Figure 3h shows that the presence of H_2_O_2_ results in a significant increase in cathodic current, which corresponds to H_2_O_2_ reduction at ~ 80 mV versus Ag/AgCl. In addition, an amperometric technique was performed to investigate the electrocatalytic reduction of H_2_O_2_ on the cathode. Figure S1 depicts the cathodic response with successive addition of H_2_O_2_. The corresponding calibration plot shows a good linearity in the range of 0.25–10 mM with the sensitivity of 9.76 μA mM^−1^. The results clearly demonstrate that the PB/MWCNT-based electrode can be efficient in reducing H_2_O_2_ in a BFC cathode or a conventional peroxide-based biosensor.

Furthermore, an amperometric technique was used to evaluate the catalytic current of the biocathode which was immobilized with GOx. Figure 3i shows that the reduction current increased upon the increase of glucose concentrations from 0.1 to 50 mM. The increase in cathodic current corresponds to the generated H_2_O_2_ (i.e., a by-product of glucose oxidation), reduced by the PB electrocatalyst in the biocathode ink’s nanocomposite. The calibration plot of a single biocathode shows a good linearity in a range of 1.5–20 mM glucose (*R*^2^ = 0.9645), as shown in Fig. 3j. The sensitivity of analysis assessed from the linear range was 0.4738 μA mM^−1^. The $K_{m}^{{{\text{app}}}}$ value on the biocathode was 2.99 mM (interpreted from the double reciprocal plot (Fig. 3k)). This was comparable with the report of a PB/MWCNT-modified electrode for glucose detection [43]. The $K_{m}^{{{\text{app}}}}$ value of the biocathode was not significantly different from that of the bioanode; consequently, the proposed enzymatic BFCs could provide a high GOx affinity toward glucose.

###  Electrochemical Reaction Kinetics of the Flexible and Printed Electrodes

To understand the additional charge-storage behavior and electrochemical reaction kinetics of the anode and the cathode, we further analyzed the CVs curves from Figs. 2c and 3f. It is obvious from the CVs that both the NQ/MWCNT-based anode and the PB/MWCNT-based cathode showed a definite redox peak, showing that redox plays a major role in capacitance. As current varies depending on different scan rates, the charge stored by the Faradaic diffusion-controlled or capacitive contributions can be determined by a *b*-value [44], which can be derived from the power law equation, see the Supporting Information Note 1. The *b*-values of the anode are 0.58 for anodic and cathodic currents, indicating the operating mechanism is dominated by a semi-infinite diffusion process (Fig. S2a), see the Supporting Information Note 1 for *b*-values derivation. This corresponds to the earlier discussion in Fig. 2d which displays that the current risen proportionately with $v^{1/2}$ due to a diffusion-controlled process at the inner nanostructured surface. The *b*-values of the cathode are 0.75 and 0.68 for anodic and cathodic current, respectively, indicating the operating mechanism is mainly dominated by diffusion (Fig. S3a).

Furthermore, the CVs curve from Figs. 2c and 3f was analyzed to find the capacitive contribution. The areal capacitance $\left( {C_{{{\text{areal}}}} } \right)$ of NQ/MWCNT-based anode and PB/MWCNT-based cathode from CVs can be estimated, see the Supporting Information Note 2. Figures S2b and S3b shows the areal capacitance of NQ/MWCNT-based anode and PB/MWCNT-based cathode as a function of the scan rate. Both electrodes had the highest areal capacitance at the low scan rate (10 mV s^−1^) and the lowest areal capacitance at the high scan rate (200 mV s^−1^). This is because low scan rates allow the electrolyte ions to diffuse into internal microscopic pores of the printed electrode, which leads to high capacitance at the electrolyte-nanocomposite interface. Capacitance decreases at high scan rates due to slower charge/discharge characteristics. However, the areal capacitance of the anode was higher than the cathode and seemed to be stable after scan rate of 100 mV s^−1^. The capacitance retention rates were 21.8% and 63.4% at scan rate of 200 mV s^−1^ for anode and cathode, respectively. The higher areal capacitance of the anode suggested that nanostructure of its ink’s components (MWCNTs network linked within graphite sheets, as shown in Fig. 2a) served as a highly conductive matrix for fast electron transport, while the lower areal capacitance of the cathode might probably be due to bigger size of PB nanoparticles on graphite sheets as shown in Fig. 3a.

To distinguish the faradaic and capacitive charge-storage contributions on each electrode, the CVs curves from Figs. 2c and 3f was analyzed followed the relation that overall current can be expressed as sum of capacitive current and faradaic current, see the Supporting Information Note 3 for details. The current response of a CV can be partitioned into faradaic and capacitive charge storage, as can be seen in Figs. S2c and S3c for the NQ/MWCNT-based anode and the PB/MWCNT-based cathode, respectively, at the scan rate of 10 mV s^−1^. In the pink region, the contribution of faradaic current is assessed, while capacitive current is estimated in the green region. At the low scan rate (10 mV s^−1^), it is evident that the faradaic charge-storage mechanism (diffusion-limited process) predominates over the capacitive charge-storage mechanism. We investigated further to a fast scan rate of 200 mV s^−1^ and discovered that the capacitive current was more dominant at a higher scan rate because diffusion limitation hinders the ability for electrolyte ions to diffuse into electrode inner layer (Figs. S2d and S3d). This clearly aligns with the areal capacitance trend observed in Figs. S2b and S3b. The presence of PB/MWCNT hybrid has a relatively important contribution to capacitive charge-storage characteristics.

###  Studies of the BFC and the Self-powered Glucose Biosensor

Glucose was selected as a biofuel model in our BFC development because it is one of the primary constituents in human biofluids, including sweat, and glucose is specifically oxidized using commercially available glucose oxidase (GOx) in catalytic electrochemical reactions, converting chemical energy to electrical energy [4, 45]. In general, the level of glucose in blood can indicate diabetes, a chronic condition caused by an insulin insufficiency [46]. However, traditional sensors for glucose detection rely on invasive blood sample methods which are painful and inconvenient. Interestingly, glucose monitoring reveals a link between glucose levels in diabetic patients’ blood and sweat [47, 48]. Therefore, researchers are exploring noninvasive sensors as glucose monitoring diagnostic tools [49]. We also aim to apply this glucose BFC-based device serving as an energy harvester to display self-powered glucose biosensing signal by investigating the relationship between glucose concentration and electrical outputs.

**Fig. 4:**
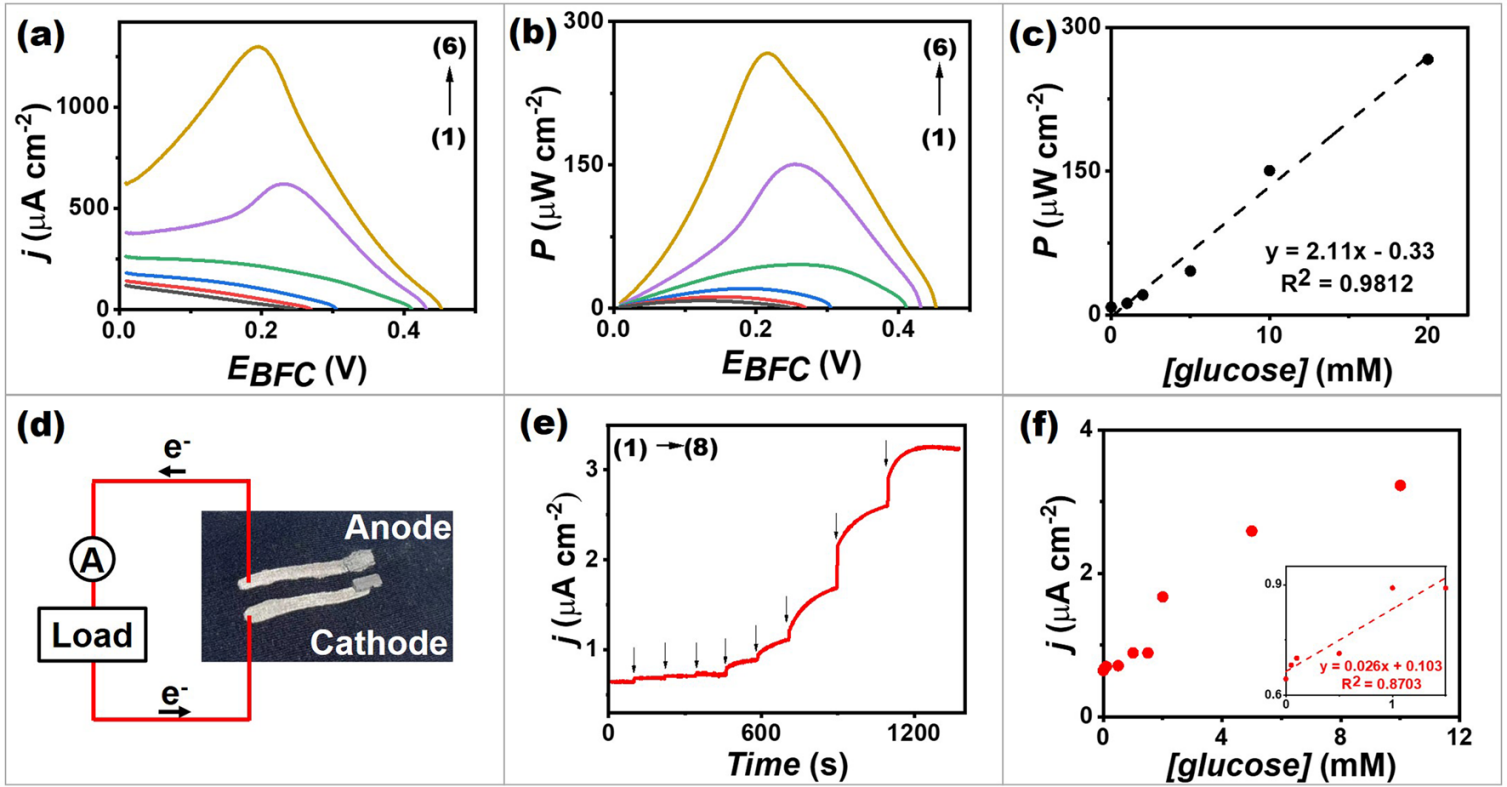
A BFC and a self-powered glucose biosensor. **a** Polarization curves of a screen-printed glucose BFC at different glucose concentrations ((1) blank; (2) 1 mM; (3) 2 mM; (4) 5 mM; (5) 10 mM; and (6) 20 mM) in 0.1 M PBS, pH 7.0.** b** Power density versus potential plots of a screen-printed glucose BFC at different glucose concentrations (1) blank; (2) 1 mM; (3) 2 mM; (4) 5 mM; (5) 10 mM; and (6) 20 mM) in 0.1 M PBS, pH 7.0. **c** The corresponding calibration plot of power and glucose concentrations. **d** Schematic illustration of the self-powered biosensing electronic system. **e** The self-generated current response obtained from a screen-printed glucose BFC, with no applied potential upon increasing the glucose concentrations (0, 0.05, 0.1, 0.5, 1, 1.5, 2, 5 and 10 mM) in 0.1 M PBS, pH 7.0. The current was observed at a constant load between electrodes of 99.7 kΩ. **f** The corresponding calibration plot of the current response and glucose concentrations of a screen-printed glucose BFC

The performance of a screen-printed glucose BFC was evaluated by using the GOx/NQ/MWCNT-based bioanode coupled with the GOx/PB/MWCNT-based biocathode in different glucose concentrations. Figure 4a and b shows that glucose concentration affects both current density and power output. The maximum current density and power output obtained reached around 1.3 mA cm^−2^ and 266 μW cm^−2^, respectively, at a voltage of 0.20 V. An open circuit voltage of 0.45 V was obtained in the presence of 20 mM glucose. This power output was reasonable for future applications of a DC–DC converter or low-power consumption electronics that could communicate wirelessly through radio frequency (RF) circuits [50]. The high current density and high-power density were obtained because of the customized nanocomposite inks in both anode and cathode, which have desirable electrical conductivity and electrocatalytic properties. MWCNTs and PB/MWCNT hybrid make it easier for electrons to pass from the anode to the cathode, leading to the high power output [21, 51]. Figure 4c shows a good relationship between the increasing concentrations of glucose and power density in a range of 0–20 mM glucose, with a good sensitivity (2.1 ± 0.1 μW cm^−2^ mM^−1^) and a correlation coefficient of 0.9812.

The screen-printed glucose BFC was investigated as a self-powered glucose biosensor. An external load was connected to BFC without the requirement of any external applied voltage, as illustrated in Fig. 4d. The current response generated by the BFC itself with successive additions of glucose in a range of 0–10 mM was observed (Fig. 4e, f). The calibration plot of the self-generated current signal showed a linearity up to 1.5 mM glucose (*R*^2^ = 0.8703), with a sensitivity of 0.026 ± 0.004 μA cm^−2^ mM^−1^. The good sensitivity in one flexible and simplified cell enables the BFC to function in a self-powered mode and be used to measure glucose concentration. The comparison between our screen-printed glucose BFC and other glucose BFC is also demonstrated in Table S2. Moreover, taking other BFCs into consideration, when BFCs are integrated with the use of other costly enzymes such as laccase [52] and bilirubin oxidase [53], it is clear that the cost of BFC fabrication appears to be higher. In addition, several of them have not been evaluated in sweat or biofluid models [7]. Additionally, the electrochemical measurements of previous published studies were tested only using a rigid electrode [10]; the ability to integrate flexible materials was not much studied.

### Selectivity Study of the Self-powered Biosensor 

**Fig. 5:**
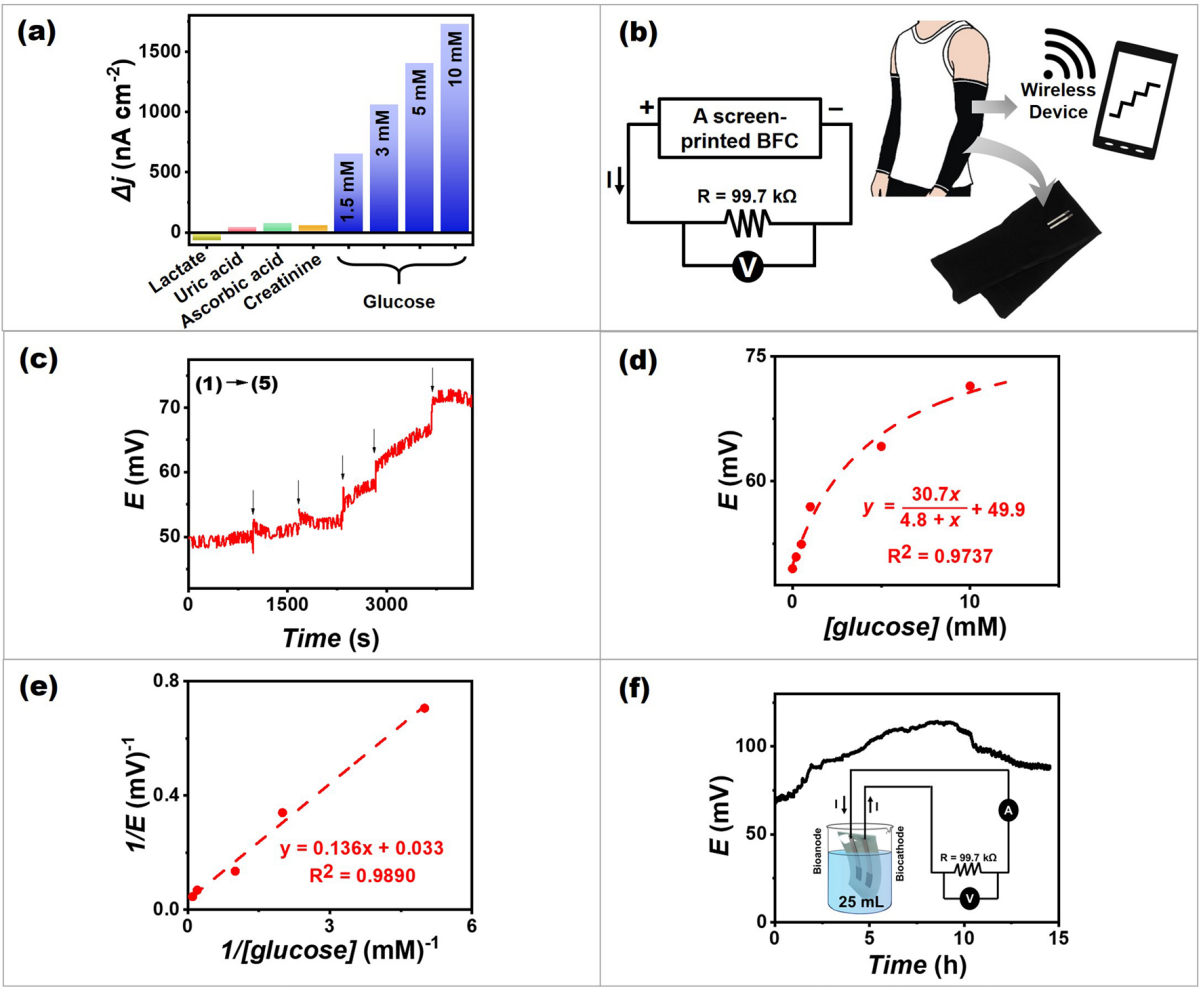
Applications of a flexible single-enzyme-based energy-harvesting device and self-powered biosensors. **a** The self-generated current response obtained from a screen-printed glucose BFC, with no applied potential upon interferences adding (14 mM lactate, 59 μM uric acid, 10 μM ascorbic acid, and 84 μM creatinine). The current was observed at a constant load between electrodes of 99.7 kΩ. **b** Schematic illustration of a screen-printed glucose BFC on a stretchable textile of the arm sleeve together with a circuit of the system. **c** The self-generated voltage output obtained from a screen-printed glucose BFC and a wireless voltmeter, with no applied potential upon increasing the glucose concentrations (0, 0.2, 0.5, 1, 5 and 10 mM) in artificial sweat. The voltage was observed at a constant load between electrodes of 99.7 kΩ. **d** The corresponding calibration plot of self-generated voltage output and glucose concentrations. **e** The double reciprocal plot of the calibration curve (background subtraction). **f** The operational stability of a screen-printed glucose BFC in the presence of 5 mM glucose in artificial sweat over 15 h with a schematic illustration of the testing cell and a circuitry system

The selectivity of a screen-printed glucose BFC was investigated by measuring the self-generated current output in the presence of the normal level of common constituents available in human sweat that might be anticipated to interfere to the determination of glucose [54]. As shown in Fig. 5a, a screen-printed glucose BFC provided high selectivity in the presence of lactate, uric acid, ascorbic acid, and creatinine. The self-generated current output shows only a slight change when interferents were added compared to the addition of the glucose target to the wearable system because of the specificity of GOx-based BFC to glucose and no extra application of any overvoltage.

Wireless technology is important for the communication between sensor units and users since it allows to access real-time digital information. Using a wireless voltmeter, the voltage output of BFCs can be transformed into an analytical signal that can be transmitted wirelessly to portable devices such as a smartphone, thereby meeting the practical requirements of their real-life wearable operation. To demonstrate this concept of a wearable and flexible biosensor, the BFC was printed on an arm sleeve and designed to measure sweat on-body, obtaining real-time data via a wireless device. The system includes a screen-printed BFC, a resistor as a load, and the compact wireless device for voltage output measuring (Fig. 5b). The voltage response obtained from a portable wireless system generated by the BFC itself with successive additions of glucose in a range of 0–10 mM in artificial sweat is illustrated in Fig. 5c. Nonlinear regression algorithms have been used to generate the calibration plot of the self-generated voltage output by using the modified Michaelis–Menten equation (Supporting Information Note 4). The calibration plot of the self-generated voltage output showed a good relationship with the increasing glucose concentration over a range of 0–10 mM glucose, with *R*^2^ = 0.9737 (Fig. 5d). This curve shows that self-generated voltage increases rapidly during the initial phase of glucose (fuel) addition, then slowly before reaching their limit. The maximum potential was attained at high glucose (substrate) concentrations due to enzyme saturation, which means that it is entirely in enzyme–substrate complex form and that adding glucose has no effect on the voltage generation. The maximum voltage obtained from the nonlinear calibration plot was comparable to the value obtained from the double reciprocal plot’s regression, which gives a good linear relationship result (Fig. 5e).

In wearable applications, the long-term and operational stabilities are of particular concern. Continuous operational stability was investigated in a batch system containing glucose in artificial sweat. The BFC showed an increase of self-generated voltage in the very first hours followed by a slow decrease after 10th hour (Fig. 5f). The enhanced voltage output observed during the initial period may reflect the H_2_O_2_ accumulation in the testing cell. These voltage outputs suggest that the BFC was still functional after long-term operation.

### Mechanical Resiliency

**Fig. 6:**
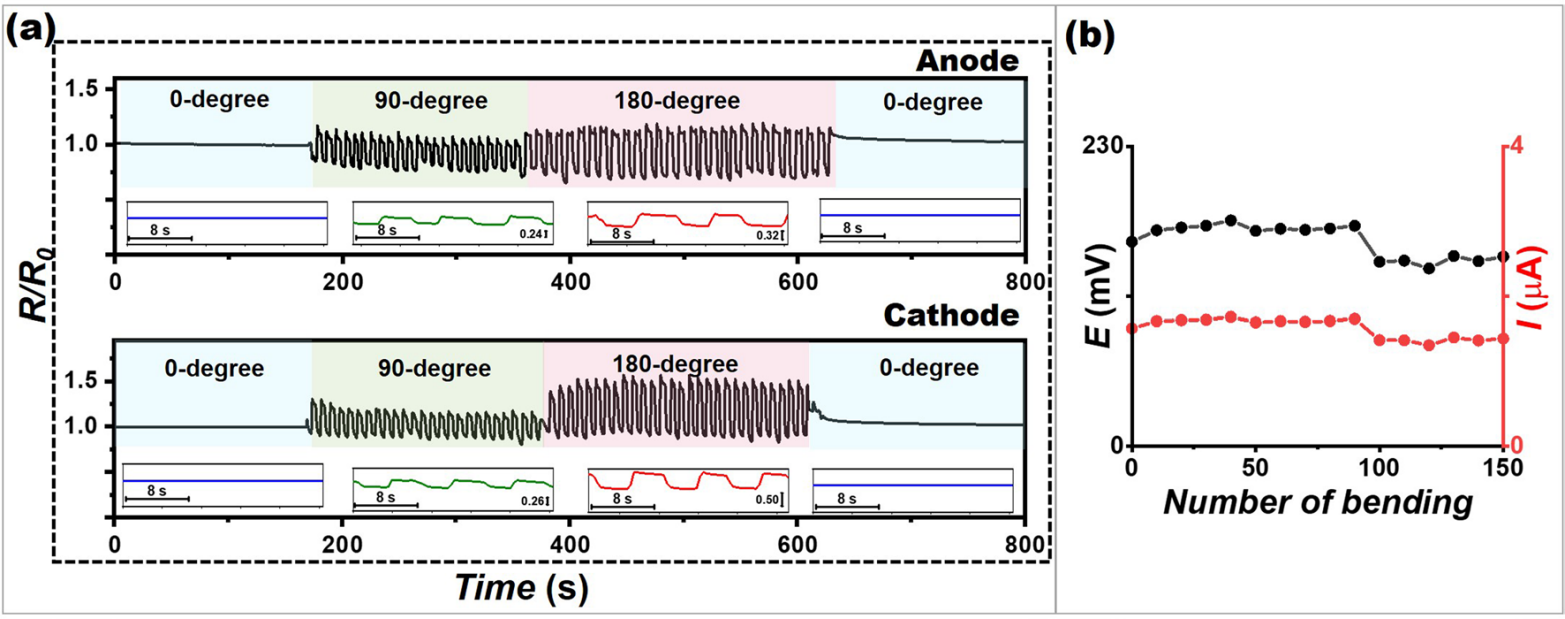
Electrical and electrochemical endurance of the printed device under multiple deformations. **a** The repetitive mechanical deformations of a screen-printed anode and cathode on PET material at 0°, 90°, 180°, and 0° bending. **b** The voltage output and current obtained from a screen-printed glucose BFC with no applied potential in the presence of 5 mM glucose in artificial sweat after a series of deformation at 180° bending. The voltage was observed at a constant load between electrodes of 99.7 kΩ

It is important for printed materials to be resilient to mechanical deformations to meet their applicability. Therefore, the effect of mechanical bending was investigated using the electrodes screen-printed on a PET material. Figure 6a shows the variation of resistance of a screen-printed anode and cathode after being bent repeatedly from 0° to 90° to 180° and back to their relaxation state at a rate of 8 s per bending cycle. When the electrode was bent from 0° to 90°, the resistance change ($R/R_{0}$) value increased slightly from the initial value, varying in a range of 0.9–1.1 for the anode and the cathode. The resistance increased with the bending degree; $R/R_{0}$ ranging between 0.8 and 1.2 for the anode the cathode at 180°. However, this occurred only during bending; after relaxing to 0°, the resistance returned to its initial value. To further confirm the ability of the BFC in practice use scenario, it was repeatedly carried out electrochemical endurance evaluation under multiple bending at 180° for 150 times in the presence of glucose in artificial sweat (Fig. 6b). Potential and current response generated by the BFC itself remained constant up to 90 times of bending, followed by maintaining over 83% performance after 100 bending cycles. After repetitive mechanical deformations, no substantial fatigue failure was observed for electrical and electrochemical aspects. This could be because when the electrode is bent, the distance between the conductive particles (MWNCTs and graphite) changes on a microscopic scale and the distance between the conductive particles increases (a larger gap between the conductive materials), thereby increasing the resistance of the electrode. However, after relaxing, elastomer polymer (i.e., PU) can assist in reestablishing the percolation network of conductive particles, facilitating the electrical current to move through the electrode. This indicates that our screen-printable inks have highly stable performances and mechanical resilience, suitable for wearable and various applications.

## Conclusion

This work demonstrated an example of screen-printable functional inks developed for a membraneless single-enzyme-based energy-harvesting device and a self-powered biosensor, powered by the same substrate (glucose) for both electrodes. The bioanode reaction relied on glucose oxidation, whereas the biocathode relied on the reduction of H_2_O_2_. The single-enzyme BFC exhibited an OCV of 0.45 V with a maximum power density of 266 μW cm^−2^ and current density of ~ 1.3 mA cm^−2^ at 20 mM glucose. The BFC was successfully used to harvest energy from artificial sweat as a representative of epidermal energy-harvesting and a self-powered glucose biosensor in real-life situations. In addition, the system had good operational stability for many hours. By creating novel screen-printable inks, the sensor enables the incorporation of a wide variety of substrates, including rubber, plastic, epidermal tattoo, and stretchable textile and the mechanical resiliency was good for electrodes printed on PET (as a representative flexible substrate). These characteristics hold great promise for on-body, noninvasive, self-powered biosensing, and energy-harvesting applications. Further study could focus on the functionality and endurance of inks screen-printed on other different substrates. Scaling up the ink preparation process for industrialization is also important to make this idea realistic for the next generation of flexible bioelectronics. It is possible to leverage this device by integrating with other on-body or wireless bioelectronics to track motion and monitor others biochemicals. Moreover, it is possible to leverage the design of energy-efficient microsystems by focusing on the design of RF circuits, power converters, and sensor interfaces for miniaturized systems and biomedical applications.

### Supplementary Information

Below is the link to the electronic supplementary material.

Supplementary file1 (DOCX 268 KB)
