# Supplementary material for: Screen-Printable Functional Nanomaterials for Flexible and Wearable Single-Enzyme-Based Energy-Harvesting and Self-Powered Biosensing Devices
Source: Nanomicro Lett. 2023 Mar 31;15:85. doi: 10.1007/s40820-023-01045-1 (PMC10066049; doi:10.1007/s40820-023-01045-1)
Supplement: Supplementary file 1 — Supplementary file1 (DOCX 268 KB) [file 40820_2023_1045_MOESM1_ESM.docx]

Supporting Information for

**Screen-Printable Functional Nanomaterials for Flexible and Wearable Single-Enzyme-Based Energy-Harvesting and Self-Powered Biosensing Devices**

Kornautchaya Veenuttranon^1^, Kanyawee Kaewpradub^1,2^ and Itthipon Jeerapan^1,2,3*^

^1^ Center of Excellence for Trace Analysis and Biosensor, Prince of Songkla University, Hat Yai, Songkhla 90110, Thailand

^2^ Division of Physical Science, Faculty of Science, Prince of Songkla University,
Hat Yai, Songkhla 90110, Thailand

^3^ Center of Excellence for Innovation in Chemistry, Faculty of Science, Prince of Songkla University, Hat Yai, Songkhla 90110, Thailand

^*^ Corresponding author: E-mail: [itthipon.j@psu.ac.th](mailto:itthipon.j@psu.ac.th) (Itthipon Jeerapan)

ORCID: <https://orcid.org/0000-0001-8016-6411> (Itthipon Jeerapan)

**S1 Supplementary Experimental Section**

# S1.1 Chemicals and Materials

Multiwalled carbon nanotubes (MWCNTs) (95% purity, diameter = 5–15 nm, length = 10–30 μm) were from Luoyang advanced material Co., Ltd. D-(+)-glucose anhydrous was from Fluka. Graphite (particle size <20 μm), glucose oxidase (GOx, from *Aspergillus niger*, type VII), 1,4–naphthoquinone (NQ), chitosan, lactic acid (C_3_H_6_O_3_), uric acid (C_5_H_4_N_4_O_3_), creatinine (C_4_H_7_N_3_O), creatine monohydrate (C_4_H_9_N_3_O_2_·H_2_O) and L-ascorbic acid (C_6_H_8_O_6_) were from Sigma-Aldrich. Urea (CH_4_N_2_O) were from Riedel-deHaën. Potassium phosphate dibasic (K_2_HPO_4_), potassium phosphate monobasic (KH_2_PO_4_), and potassium ferricyanide (K_3_Fe(CN)_6_) were from BDH Chemicals Ltd. Potassium chloride (KCl) was from Rankem, RFCL Ltd. Ferric chloride anhydrous (FeCl_3_), hydrogen peroxide 30% (H_2_O_2_), and sodium chloride (NaCl) were from Merck. Sodium carbonate (Na_2_CO_3_) and sodium phosphate monobasic (NaH_2_PO_4_·2H_2_O) were from Ajax Finechem. Di-Sodium hydrogen phosphate 12-hydrate (Na_2_HPO_4_·12H_2_O) was from KemAus. Tetrahydrofuran (THF) was from Honeywell, B&J brand. Hydrochloric acid (HCl) was from RCI Labscan Ltd. Polyurethane (PU) (Tecoflex® SG-80A) was from Lubrizol Life Sciences. All chemical solutions were prepared using ultrapure deionized water (18.2 MΩ cm) from a Milli Q Merck system (Germany). The phosphate buffered solution (PBS) (0.1 M) was made with a pH value of 7.0. Glucose solution was prepared before use for at least 24 h and stored at 4 °C.

# S1.2 Preparation of Artificial Sweat

The compositions described in the literature were followed for preparing an artificial sweat solution with a modification [S[1](#_ENREF_1)]. Briefly, 0.2 M phosphate-buffered saline was first prepared. Specifically, 71.60 g L^–1^ of Na_2_HPO_4_⋅12H_2_O and 7.24 g L^–1^ of NaH_2_PO_4_·2H_2_O were separately prepared, and named as stock A and B, respectively. Solutions A and B were mixed at the ratio of 40.5 : 9.5 mL to obtain 50 mL of phosphate-buffered saline solution. Then, 0.45 g NaCl was added. Lactic acid, uric acid, urea, creatinine, creatine, and ascorbic acid were added to the PBS solution according to the median values of human sweat constituents stated in Table S3 [S[2](#_ENREF_2)], resulting in artificial sweat.

# S1.3 Preparation of Inks

## *S1.3.1 Synthesis of PB/MWCNT Nanocomposite*

PB-based nanocomposite was prepared to enhance peroxide catalytic activity on the biocathode. One-pot preparation of PB/MWCNT was conducted as follows, with a modification [S[3](#_ENREF_3)]. First, 45 mg of MWCNTs was dispersed in 15 mL of water using a homogenizer probe (model AR-0975) level 1 for 90 s. Next, 15 mL of MWCNT dispersion were added to 300 mL of the acidic mixture (pH 1.5, adjusted with HCl) of Fe^3+^ (containing 603 mg FeCl_3_, 764 mg K_3_Fe(CN)_6_, and 3,348 mg KCl) with vigorous stirring. The suspension was stirred for 24 h, and the resulting solid of the PB/MWCNT nanocomposite was obtained by centrifugation (9,000 rpm, 5 min) and excess ions were removed thoroughly with water two times. The PB/MWCNT particles were then left to dry in a hot air oven at 40 °C for 24 h.

## *S1.3.2 Anode NQ/MWCNT-based Ink*

The NQ/MWCNT-based ink was prepared by dispersing 100 mg of MWCNTs in 3 mL of THF using a homogenizer probe, level 1 for 90 s. Simultaneously, 50 mg of NQ was dissolved in 0.3 mL of THF with vigorous shaking. Then, 1,000 mg of graphite, 105 mg of PU (dissolved in 0.7 mL of THF), dissolved NQ, and dispersed MWCNTs were mixed using a mixing machine (Shashin Kagaku, Kakuhunter, SK-300SII, Japan) at 1,500 rpm for 5 min. For anode preparation, the NQ/MWCNT-based ink was printed on the substrate with controlling the solid-to-solvent ratio of 1 mg : 4 μL.

## *S1.3.3 Cathode PB/MWCNT-based Ink*

PB/MWCNT-based ink was prepared by dispersing 100 mg of as-prepared PB/MWCNT powder in 1 mL of THF using a homogenizer probe, level 1 for 90 s. Then, 1,000 mg of graphite and 105 mg of PU (dissolved in 0.7 mL THF) were mixed with the mixture using a mixing machine at 1,500 rpm for 5 min. For cathode preparation, the PB/MWCNT-based ink was printed on the substrate with controlling the solid-to-solvent ratio of 1 mg : 2.7 μL.

## S1.4 Preparation of an Enzymatic BFC

For modifying a bioanode, the printed NQ/MWCNT-based electrode was first cleaned by applying a constant voltage of 1.20 V in 1.0 M Na_2_CO_3_ for 60 s, and then followed by drop casting 8 μL of 30 mg mL^–­­­­­1^ GOx in 0.10 M PBS, pH 7.4. After air drying, 6 μL of 1.5% (w/v) chitosan (in 0.25 M acetic acid) was dropped to cover electrode surface. For modifying a biocathode, the printed PB/MWCNT-based electrode was functionalized by drop-casting 8 μL of 30 mg mL^–­­­­­1^ GOx. After air drying, 6 μL of 1.5% (w/v) chitosan was dropped. The BFCs were left to dry for 24 h at 4 °C.

## S1.5 Microscopic and Functional Group Characterizations

Scanning electron microscope (SEM) was used to characterize the microscopic morphology of electrodes. EDX mappings for the cathode were performed with energy-dispersive spectrometer mounted on the SEM. Fourier transform infrared (FT-IR) spectra were recorded in the range of 400−4000 cm^−1^ by loading the samples (MWCNT and PB/MWCNT) in a KBr pellet on a Nicolet™ iS™ 5 FT-IR spectrometer.

## S1.6 Resistance and Electrochemical Measurement

The resistance and short circuit current output in the self-powered mode were measured using a digital multimeter (6½ digit Keysight, model 34465A) and the data was recorded through a Keysight BenchVue Software (version 3.0). Electrochemical experiments, i.e., cyclic voltammetry (CV), amperometry, and linear sweep voltammetry (LSV), were performed using a μAutolab Type III FRA 2 (Metrohm) controlled by NOVA software. To perform experiments in a three-electrode system, our developed electrodes were evaluated with a Pt wire and Ag/AgCl (in 3.0 M KCl) as counter and reference electrodes. A glassy carbon electrode (GC) was only used as a working electrode in PB/MWCNT characterization. All experiments were carried out at room temperature and in 0.1 M PBS, pH 7.0, unless otherwise specified.

## S1.7 Self-powered Detection of Glucose

For the BFC characterization, a two-electrode system was carried out. The open circuit voltage (OCV) was monitored before and after the addition of glucose solution. The polarization curve was obtained from OCV to 0 V with a scan rate of 5 mV s^–1^. The self-powered current output was recorded through a digital multimeter with an external load of 99.7 kΩ while voltage and current were measured by using a compact voltmeter (Vernier GO Direct® Electrode Amplifier with an internal lithium-ion rechargeable battery), and a digital multimeter. The selectivity of a screen-printed glucose BFC was investigated in solutions containing common constituents available in human sweat. Specifically, the self-generated current was first investigated in 0.10 M PBS, pH 7.0 containing 1.5 mM glucose, followed by successively testing with interferences (14 mM lactate, 59 μM uric acid, 10 μM ascorbic acid, and 84 μM creatinine), containing 1.5 mM glucose, and ending up with glucose (3, 5, and 10 mM).

**S2 Supplementary Figures**


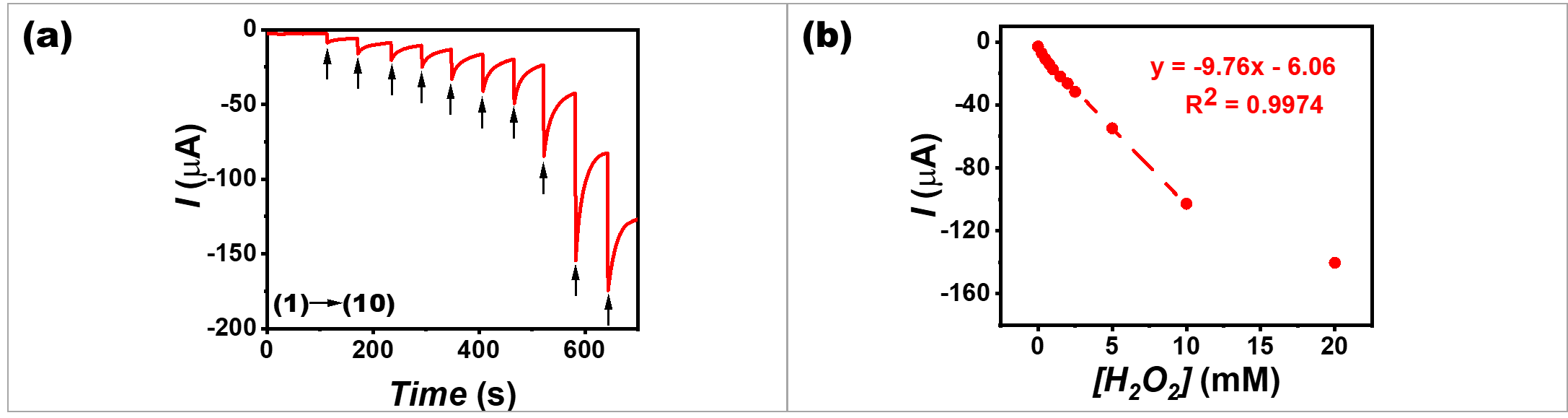


**Fig. S1** Electrochemical study of the flexible PB/MWCNT-based cathode toward electrocatalytic H_2_O_2_ reduction. **a** Amperometric response of a screen-printed PB/MWCNT-based cathode versus Ag/AgCl as a reference electrode and Pt as a counter electrode, with an applied potential of 0 V upon increasing the H_2_O_2_ concentrations ((1) 0.25 mM; (2) 0.50 mM; (3) 0.75 mM; (4) 1.00 mM; (5) 1.50 mM; (6) 2.00 mM; (7) 2.50 mM; (8) 5.00 mM; (9) 10.00 mM; and (10) 20.00 mM). **b** The corresponding calibration plot of the current response of the PB/MWCNT-based cathode


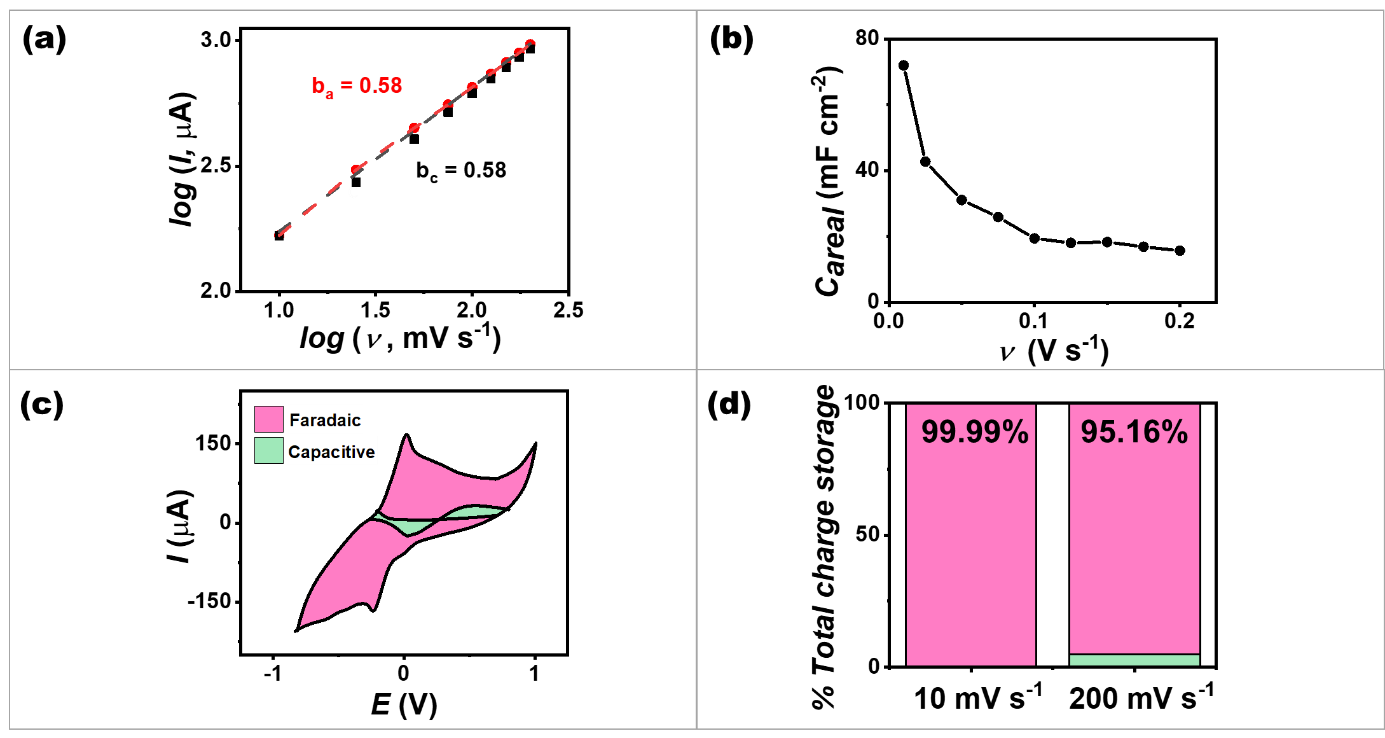


**Fig. S2** Electrochemical reaction kinetics of a screen-printed NQ/MWCNT-based anode. **a** *b-*value determination of the anodic and cathodic peak currents. **b** Areal capacitance variations at different scan rates from 10–200 mV s^−1^. **c** Charge storage contributions in the NQ/MWCNT-based anode at 10 mV s^−1^. **d** The breakdown for charge storage contributions (capacitive and faradaic)


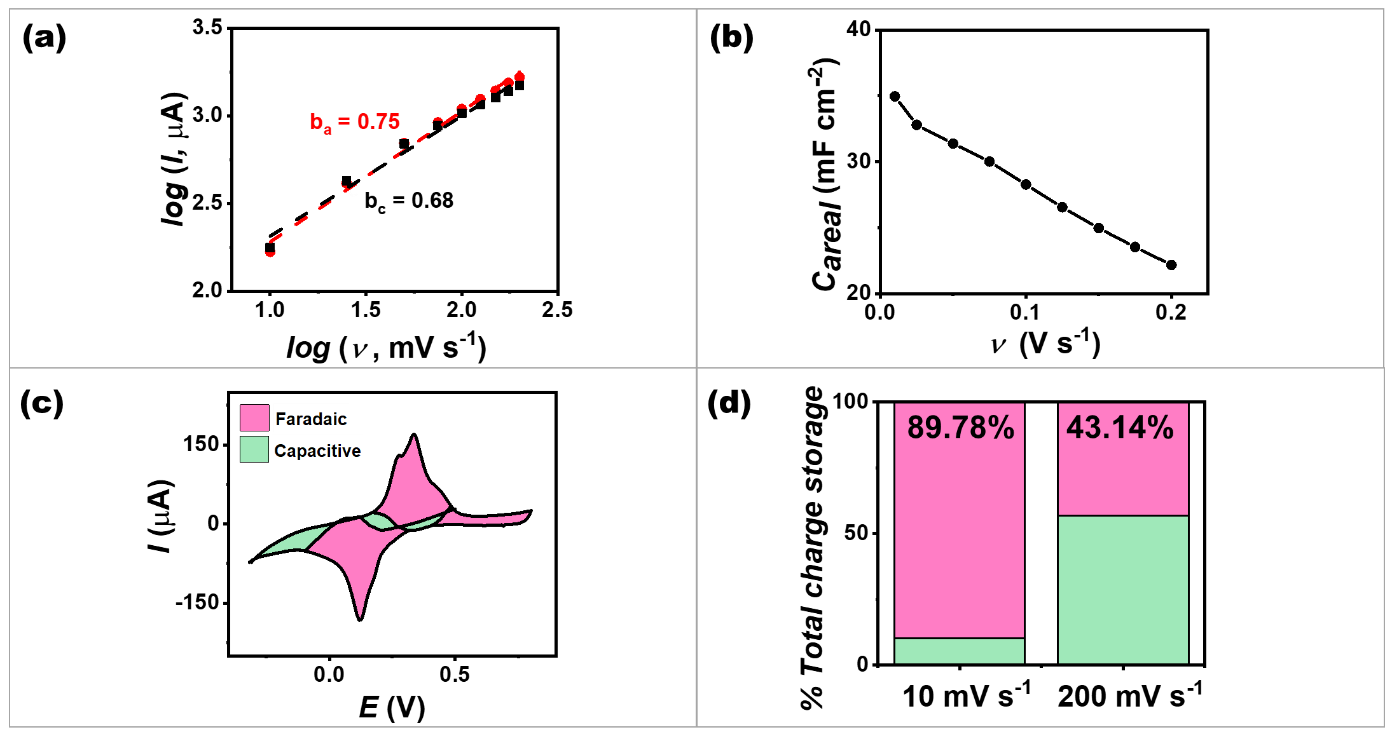


**Fig. S3** Electrochemical reaction kinetics of a screen-printed PB/MWCNT-based cathode. **a** *b-*value determination of the anodic and cathodic peak currents. **b** Areal capacitance variations at different scan rates from 10–200 mV s^−1^. **c** Charge storage contributions in the PB/MWCNT-based cathode at 10 mV s^−1^. **d** The breakdown for charge storage contributions (capacitive and faradaic)

**S3 Supplementary Tables**

**Table S1** Comparison of different GOx-based BFCs using glucose on the bioanode and the biocathode

| Fuel(s) on the anode and the cathode | Electrodes | Detection ranges | OCV (V) | Power  (μW cm^-2^) | Electrolyte | Screen printing | Flexible | Ref. |
| --- | --- | --- | --- | --- | --- | --- | --- | --- |
| glucose and H_2_O_2_ generated by glucose | GOx/5–Amino-1,10–Phenanthroline/graphite rod bioanode and GOx/horseradish peroxidase/graphite rod biocathode | 0–150 mM glucose | 0.45 | 3.5 | 0.05 M sodium acetate-sodium phosphate buffer, pH 6.0 (containing 0.1 M KCl) | ✗ | ✗ | [S[4](#_ENREF_4)] |
| glucose and H_2_O_2_ generated by glucose | GOx/poly(pyrrole–2–carboxylic acid)/gold nanoparticles/poly(1,10–phenanthroline–5,6–dione)/graphite rod bioanode and GOx/poly(pyrrole–2–carboxylic acid)/Prussian blue/graphite rod biocathode | 0.01–195 mM glucose | 0.64 | 10.9 | O_2_-saturated sodium acetate-sodium phosphate -KCl buffer solution, pH 6.0 | ✗ | ✗ | [S[5](#_ENREF_5)] |
| glucose and H_2_O_2_ generated by glucose | chitosan/GOx/NQ/MWCNT/ graphite/silver bioanode and chitosan/GOx/Prussian blue /MWCNT/ graphite/silver biocathode | 0.05–10 mM glucose | 0.45 | 266.0 | 0.1 M phosphate buffer solution, pH 7.0 / artificial sweat | ✓ | ✓ | This work |

**Table S2** Comparison of glucose BFCs

| Fuel(s) on the anode and the cathode | Electrodes | Glucose ranges | OCV (V) | Power  (μW cm^-2^) | Electrolyte | Screen printing | Flexible | Ref. |
| --- | --- | --- | --- | --- | --- | --- | --- | --- |
| glucose and H_2_O_2_ directly converted by GOx | GOx/5–Amino-1,10–Phenanthroline/graphite rod bioanode and GOx/horseradish peroxidase/graphite rod biocathode | 0–150 mM glucose | 0.45 | 3.5 | 0.05 M sodium acetate-sodium phosphate buffer, pH6.0 (containing 0.1 M KCl) | ✗ | ✗ | [S[4](#_ENREF_4)] |
| glucose and H_2_O_2_ directly converted by GOx | GOx/poly(pyrrole–2–carboxylic acid)/gold nanoparticles/poly(1,10–phenanthroline–5,6–dione)/graphite rod bioanode and GOx/poly(pyrrole–2–carboxylic acid)/Prussian blue/graphite rod biocathode | 0.01–195 mM glucose | 0.64 | 10.9 | O_2_-saturated APS-KCl buffer solution, pH 6.0 | ✗ | ✗ | [S[5](#_ENREF_5)] |
| glucose and O_2_ | tetrathiafulvalene/GOx/carbon paste bioanode and Pt black/carbon paste cathode | 5–25 mM glucose | 0.26 | 7.0 | 0.1 M phosphate buffer solution, pH 7.4 / artificial interstitial fluid | ✗ | ✗ | [S[6](#_ENREF_6)] |

**Table S2** Comparison of glucose BFC (continue)

| Fuel(s) on the anode and the cathode | Electrodes | Glucose ranges | OCV (V) | Power  (μW cm^-2^) | Electrolyte | Screen printing | Flexible | Ref. |
| --- | --- | --- | --- | --- | --- | --- | --- | --- |
| glucose and O_2_ | flavin-adenine-dinucleotide-dependent glucose dehydrogenase/MgO-templated mesoporous carbon modified with poly(glycidyl methacrylate)/carbon paste bioanode and bilirubin oxidase/MgO-templated mesoporous carbon modified with poly(glycidyl methacrylate)/carbon paste biocathode | 0–10 mM glucose | 0.77 | - | 1 M phosphate buffer solution, pH 7.0 / artificial urine | ✓ | ✓ | [S[7](#_ENREF_7)] |
| glucose and O_2_ | glucose gdehydrogenase/pyrroloquinoline quinone/cabon bioanode and 1-pyrenebutyric acid N-hydroxysuccinimide ester/bilirubin oxidase/graphitized carbon nanofibers/carbon biocathode | 0.1–10 mM lucose | 0.57 | 6.8 | air-saturated phosphate buffer solution, pH 7.4 | ✗ | ✗ | [S[8](#_ENREF_8)] |
| glucose and O_2_ | GOx/tetrathiafulvalene/carbon/filter paper bioanode and bilirubin oxidase/carbon/filter paper biocathode | 1–25 mM glucose | 0.57 | 120 | 1 M phosphate buffer solution, pH 7.0 / artificial urine | ✓ | ✗ | [S[9](#_ENREF_9)] |

**Table S2** Comparison of glucose BFC (continue)

| Fuel(s) on the anode and the cathode | Electrodes | Detection ranges | OCV (V) | Power  (μW cm^-2^) | Electrolyte | Screen printing | Flexible | Ref. |
| --- | --- | --- | --- | --- | --- | --- | --- | --- |
| glucose and manganese oxide | GOx/cobalt phthalocyanine/1–pyrenebutyric acid/buckypaper bioanode and manganese oxide/1–pyrenebutyric acid/buckypaper | 0.5–8 mM glucose | 0.65 | 136.0 | 0.1 M phosphate buffer solution, pH 7.0 / human serum | ✗ | ✗ | [S[10](#_ENREF_10)] |
| glucose and silver oxide | GOx/NQ/CNT bioanode and silver oxide/silver cathode | 0–50 mM glucose | 0.44 | 160.0 | 0.1 M phosphate buffer solution, pH 7.0 / sweat | ✓ | ✓ | [S[11](#_ENREF_11)] |
| glucose and H_2_O_2_ directly converted by GOx | chitosan/GOx/NQ/MWCNT/graphite/silver bioanode and chitosan/GOx/PB/MWCNT/graphite/silver biocathode | 0.05–10 mM glucose | 0.45 | 266.0 | 0.1 M phosphate buffered saline, pH 7.0 / artificial sweat | ✓ | ✓ | This work |

**Table S3** Formulation of an artificial sweat [S[2](#_ENREF_2)]

| **Constituents** | **Concentration (M)** |  |
| --- | --- | --- |
|  |  |  |
| Lactic acid (C_3_H_6_O_3_) | 1.4 × 10^–2^ |  |
| Uric acid (C_5_H_4_N_4_O_3_) | 5.9 × 10^–5^ |  |
| Urea (CH_4_N_2_O) | 1.0 × 10^–2^ |  |
| Creatinine (C_4_H_7_N_3_O) | 8.4 × 10^–5^ |  |
| Creatine monohydrate (C_4_H_9_N_3_O_2_·H_2_O) | 1.5 × 10^–5^ |  |
| Ascorbic acid (C_6_H_8_O_6_) | 1.0 × 10^–5^ |  |

**Supporting Information Note 1: The *b*-value**

As current varies depending on different scan rates, the charge stored by the Faradaic diffusion-controlled or capacitive contributions can be determined by a *b*-value [S[12](#_ENREF_12)], which can be derived from the power law equation (Supporting Equation S1):

$i = {a\nu}^{b}$ (S1)

Where $i$is the current (A), $v$ is the scan rate (V s^−1^), $a$ and $b$ are arbitrary coefficients. The *b*-values can be calculated from the slope obtained by plotting the peak currents and scan rates in a log plot with an assumption that the current obeys the power-law relationship.

**Supporting Information Note 2: The areal capacitance calculations**

The areal capacitance of an electrode was estimated according to CVs data and calculated by Supporting Equation S2 and S3.

$C= \frac{1}{2 \times(V_{2}-V_{1})\times v} \int_{V_{1}}^{V_{2}} \left| I(V) \right|dV$ (S2)

$C_{areal}=\frac{C}{A}$ (S3)

Where *I(V)* is the instantaneous current in each potential (A), $V_{2}$ and $V_{1}$ are the upper and lower potential of the chosen potential window (V), $v$ is the scan rate (V s^–1^), and $A$ is the geometrical area of the printed electrode (cm^2^).

**Supporting Information Note 3: The faradaic and capacitive charge-storage contributions**

In order to distinguish the faradaic and capacitive charge-storage contributions on each electrode, the CVs curves from was analyzed followed the relation that the overall current ($i_{total}$) at a given potential can be expressed as the sum of two separate charge storage mechanisms, i.e., capacitive current ($i_{c}$) and faradaic diffusion limited current ($i_{d}$), using the Supporting Equation S4 [S[13](#_ENREF_13)].

$i_{total}=i_{c}+i_{d}=k_{c}v+k_{d}v^{1/2}$ (S4)

Where $k_{c}v$ and$k_{d}v^{1/2}$ are the current contribution from capacitive and faradaic charge storage mechanism, respectively. The $k_{c}$ and $k_{d}$ values can be calculated from the slope and y-intercept obtained by plotting the current response at each potential normalized to the square root of the scan rate, using Supporting Equation S5.

$\frac{i_{total}}{v^{1/2}}=k_{c}v^{1/2}+k_{d}$ (S5)

The estimated capacitive contribution area which exceeds the total CV curve is trimmed so that it falls solely within the total CV region.

**Supporting Information Note 4: The modified Michaelis-Menten equation**

The modified Michaelis-Menten equation for consumed substrate (glucose) is presented as:

$E= \frac{E_{max} \times[glucose]}{K_{m}+[glucose]}+C$ (S6)

Where $E$ is a generated potential after the addition of glucose, $[glucose]$ is the glucose concentration, $E_{max}$ is the maximum potential obtained from saturated glucose concentrations and *C* is a constant.

**Supplementary References**

[S1] X. Wei, M. Zhu, J. Li, L. Liu, J. Yu, Z. Li, B. Ding. Wearable biosensor for sensitive detection of uric acid in artificial sweat enabled by a fiber structured sensing interface. Nano Energy. **85**, 106031 (2021). <https://doi.org/10.1016/j.nanoen.2021.106031>

[S2] C. J. Harvey, R. F. LeBouf, A. B. Stefaniak. Formulation and stability of a novel artificial human sweat under conditions of storage and use. Toxicol. Vitro. **24**, 1790-1796 (2010). <https://doi.org/10.1016/j.tiv.2010.06.016>

[S3] J. Zhai, Y. Zhai, D. Wen, S. Dong. Prussian blue/multiwalled carbon nanotube hybrids: Synthesis, assembly and electrochemical behavior. Electroanalysis. **21**, 2207-2212 (2009). <https://doi.org/10.1002/elan.200904680>

[S4] V. Krikstolaityte, Y. Oztekin, J. Kuliesius, A. Ramanaviciene, Z. Yazicigil, M. Ersoz, A. Okumus, A. Kausaite-Minkstimiene, Z. Kilic, A. O. Solak, A. Makaraviciute, A. Ramanavicius. Biofuel cell based on anode and cathode modified by glucose oxidase. Electroanalysis. **25**, 2677-2683 (2013). <https://doi.org/10.1002/elan.201300482>

[S5] A. Kausaite-Minkstimiene, A. Kaminskas, A. Ramanaviciene. Development of a membraneless single-enzyme biofuel cell powered by glucose. Biosens. **216**, 114657 (2022). <https://doi.org/10.1016/j.bios.2022.114657>

[S6] G. Valdés-Ramírez, Y.-C. Li, J. Kim, W. Jia, A. J. Bandodkar, R. Nuñez-Flores, P. R. Miller, S.-Y. Wu, R. Narayan, J. R. Windmiller, R. Polsky, J. Wang. Microneedle-based self-powered glucose sensor. Electrochem. commun. **47**, 58-62 (2014). <https://doi.org/10.1016/j.elecom.2014.07.014>

[S7] I. Shitanda, Y. Fujimura, T. Takarada, R. Suzuki, T. Aikawa, M. Itagaki, S. Tsujimura. Self-powered diaper sensor with wireless transmitter powered by paper-based biofuel cell with urine glucose as fuel. ACS Sens. **6**, 3409-3415 (2021). <https://doi.org/10.1021/acssensors.1c01266>

[S8] P. Pinyou, F. Conzuelo, K. Sliozberg, J. Vivekananthan, A. Contin, S. Pöller, N. Plumeré, W. Schuhmann. Coupling of an enzymatic biofuel cell to an electrochemical cell for self-powered glucose sensing with optical readout. Bioelectrochemistry. **106**, 22-27 (2015). <https://doi.org/10.1016/j.bioelechem.2015.04.003>

[S9] I. Shitanda, Y. Fujimura, S. Nohara, Y. Hoshi, M. Itagaki, S. Tsujimura. Paper-based disk-type self-powered glucose biosensor based on screen-printed biofuel cell array. J. Electrochem. Soc. **166**, B1063-B1068 (2019). <https://doi.org/10.1149/2.1501912jes>

[S10] S. Hao, H. Zhang, X. Sun, J. Zhai, S. Dong. A mediator-free self-powered glucose biosensor based on a hybrid glucose/mno2 enzymatic biofuel cell. Nano Res. **14**, 707-714 (2021). <https://doi.org/10.1007/s12274-020-3101-5>

[S11] I. Jeerapan, J. R. Sempionatto, A. Pavinatto, J.-M. You, J. Wang. Stretchable biofuel cells as wearable textile-based self-powered sensors. J. Mater. Chem. A. **4**, 18342-18353 (2016). <https://doi.org/10.1039/C6TA08358G>

[S12] Z. Tian, X. Tong, G. Sheng, Y. Shao, L. Yu, V. Tung, J. Sun, R. B. Kaner, Z. Liu. Printable magnesium ion quasi-solid-state asymmetric supercapacitors for flexible solar-charging integrated units. Nat. Commun. **10**, 4913 (2019). <https://doi.org/10.1038/s41467-019-12900-4>

[S13] K. B. Hatzell, M. Boota, E. C. Kumbur, Y. Gogotsi. Flowable conducting particle networks in redox-active electrolytes for grid energy storage. J. Electrochem. Soc. **162**, A5007 (2015). <https://doi.org/10.1149/2.0011505jes>
